# Ligaments of the scapho-trapezial-trapezoidal joint: MR anatomy in asymptomatic and symptomatic individuals

**DOI:** 10.1007/s00256-021-03865-x

**Published:** 2021-07-26

**Authors:** Kai Higashigaito, Christian W. A. Pfirrmann, Sarah Koch, Dimitri Graf, Andreas Schweizer, Daniel Nanz, Andrea B. Rosskopf

**Affiliations:** 1 Radiology, Balgrist University Hospital, Forchstrasse 340, 8008 Zurich, Switzerland; 2 Institute of Diagnostic and Interventional Radiology, University Hospital Zurich, University of Zurich, Raemistrasse 100, 8091 Zurich, Switzerland; 3 MRI Medical Radiological Institute Zurich, Zurich, Switzerland; 4 Handsurgery, Balgrist University Hospital, Forchstrasse 340, 8008 Zurich, Switzerland; 5 Swiss Center for Musculoskeletal Imaging, Balgrist Campus AG, Zurich, Switzerland; 6 ARISTRA, Radiology, Zurich, Switzerland

**Keywords:** STT ligament complex, Anatomy, Wrist, MRI

## Abstract

**Purpose:**

To evaluate the MRI anatomy of the scapho-trapezial-trapezoidal (STT) ligament complex in asymptomatic and symptomatic individuals.

**Material and methods:**

In this retrospective study, STT ligament complex of 42 (male 69%, median age 37.5 years) asymptomatic (*n* = 25) and symptomatic (*n* = 17) (defined as pain described over the STT joint) individuals was examined using a high-resolution 3D proton density-weighted isovoxel sequence (MR arthrogram) with multiplanar reconstructions. Two musculoskeletal radiologists independently assessed visibility, signal intensity (SI), morphology, and thickness of the radiopalmar scapho-trapezial ligament (rpSTL), palmar scapho-capitate capsular ligament (pSCL), palmar STT capsule (pSTTC), and dorsal STT capsule (dSTTC).

**Results:**

Interreader agreement ranged from fair to good and intraclass correlations were good. The rpSTL was almost always visible (85.7%/80.1%; reader 1/reader 2). The pSCL and dSTTC were visible in all cases. The pSTTC was visible in only 52.4%/42.9%. Mean thickness of the rpSTL, pSCL, pSTTC, and dSTTC was 1.4 ± 0.5 mm/1.3 ± 0.5 mm, 2.8 ± 0.7 mm/2.7 ± 0.6 mm, 0.5 ± 0.5 mm/0.4 ± 0.4 mm, and 0.5 ± 0.3 mm/0.3 ± 0.3 mm. Both readers rated SI of the rpSTL significantly more often as increased in the symptomatic group (increased SI in asymptomatic group: 20%/15%; symptomatic group: 56%/50%) (*p*-values < 0.005). For all other ligaments, no significant difference was observed for SI between symptomatic and asymptomatic group (*p*-values ranging between 0.188 and 0.890). For all other ligaments, no significant differences were observed regarding ligament visibility, morphology, and thickness (*p*-values ranging between 0.274 and 1.000).

**Conclusion:**

The anatomy of the STT ligament complex can consistently be visualized on high-resolution 3D MRI. Increased signal intensity of rpSTL is significantly more frequent in patients with radial-sided wrist pain.

## Introduction

**Fig. 1:**
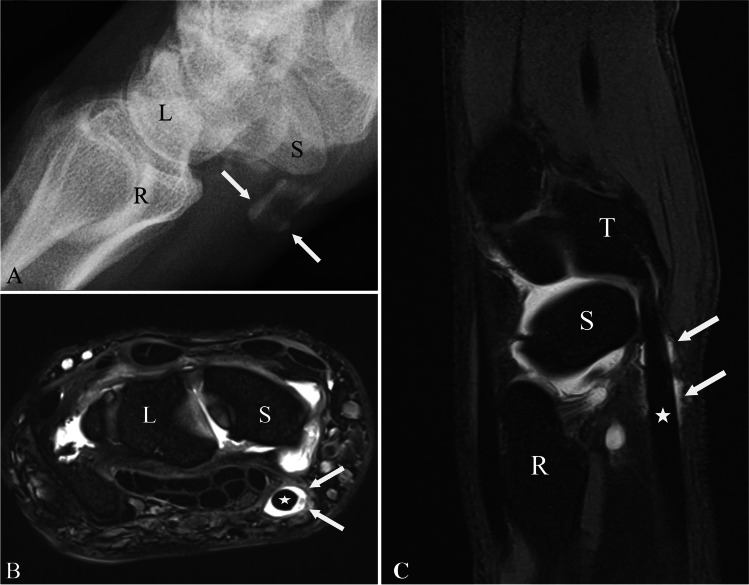
Contrast media in the tendon sheath of the flexor carpi radialis tendon. Fluoroscopic image (**a**) of contrast media in the tendon sheath of the flexor carpi radialis tendon (white arrows) after midcarpal contrast media injection as an indirect sign of a tear of the STT ligament complex. Corresponding axial (**b**) (proton density with fat saturation) and sagittal (**c**) (T1 weighted with fat saturation) MR images also show contrast media (white arrows) in the tendon sheath of the flexor carpi radialis (white star). Abbreviations: S, scaphoid; L, lunate; R, radius; T, trapezium

The scapho-trapezial-trapezoid (STT) joint is a common source of radial wrist pain. Osteoarthritis of the STT joint is very frequent. Besides osteoarthritis, posttraumatic instability of the STT may occur, which is addressed with surgery. However, the ligament complex stabilizing the STT joint almost never receives attention at imaging. It has been anecdotically described that the key radiological investigation is the arthrogram of the midcarpal joint with contrast media communication from the STT joint into the tendon sheath of the flexor carpi radialis tendon allowing the incident diagnosis of the STT ligament complex tear [1] (Fig. 1).

The stabilization of this joint depends on the surrounding ligaments, the so-called STT ligament complex. Little is known about the MRI appearance of this ligament complex [2, 3]. Anatomical studies have shown that the STT ligaments complex consists of 4 components, namely, the radiopalmar scapho-trapezial ligament (rpSTL), the palmar scapho-trapezial-trapezoidal capsule (pSTTC), the dorsal scapho-trapezial-trapezoidal capsule (dSTTC), and the scapho-capitate ligament (pSCL) [4–7]. The rpSTL has been described as the main stabilizer of the STT joint [8]. Ligaments of the wrists often measure only few millimeters and conventional 2D MRI sequences with slice thickness between 2 to 3 mm are therefore often not sufficient for the proper assessment of those ligaments. Moreover, the course of those ligaments often does not follow the standard imaging planes of the wrist (coronal, sagittal, or axial), making the identification and assessment of those ligaments even more challenging.

Advancements of magnetic resonance imaging (MRI) technology with the development of robust 3D high-resolution isovoxel sequences help to overcome these imaging limitations. Tailored reconstructions of the images in the plane of the ligament course result in better visualization and detection of these small anatomical structures in the wrist [9]. The use of 3 T MRI is recommended over 1.5 T MRI due to better image definition and additional intraarticular contrast media application further increases the sensitivity of ligament detection (better contrast amplification between structures) [3].

To the best of our knowledge, no study systematically evaluated the MRI morphology of the STT ligament complex so far. Thus, the purpose of this study was to qualitatively and quantitatively evaluate the MRI anatomy of the STT ligament complex in asymptomatic and symptomatic individuals in 3 T MR arthrograms of the wrist including a high-resolution 3D proton density-weighted sequence.

## Material and methods

This retrospective study was approved by the local ethics committee. Written informed consent was waived due to the retrospective study design. Between August 2019 and March 2020, our institutional PACS system was searched for patients with MR arthrogram of the wrist and the corresponding medical histories were reviewed. Inclusion criteria were as follows: age over 18 years, detailed documentation of pain localization. Exclusion criteria were as follows: patients with a history of previous surgery at the wrist and bad image quality. Image quality was assessed by a radiologist (blinded) with more than 10 years of experience who was not involved in the readout. All patients were examined by experienced hand surgeons. Patients having localized radial-sided wrist pain over the STT joint were categorized as symptomatic.

### MR imaging protocol

All patients had received an intraarticular injection of contrast media under fluoroscopic guidance according to our institutional standard protocol: A mixture of iodinated contrast media (Iopamiro 200 (200 mg/ml), Bracco Suisse SA, Switzerland) and gadoteric acid (Artirem (0.0025 mmol Gadolinium/ml), Guerbet AG, Zurich, Switzerland) with a volume mixing ratio of 1:2 was used. First, contrast media was injected into the distal radioulnar joint (1–2 ml) followed by the midcarpal joint (triquetral-hamate-capitate-lunate (four corner) interval) (2–4 ml).

All scans were performed using a MRI device with a magnetic field strength of 3 Tesla (Magnetom Vida, Siemens Healthineers, Forchheim, Germany). All patients were placed in a prone position, the arm extended over the head, the elbow joint in pronation and the wrist placed as close as possible to the isocenter of the magnet. A dedicated 16 channel wrist coil was used. The following sequences were acquired according to our institutional standard protocol: proton density SPACE (sampling perfection with application-optimized contrast using different flip-angle evolutions) in coronal plane (repetition time ms/echo time ms: 1000/55; flip angle 150°, voxel size 0.2 × 0.2 × 0.5 mm; turbo factor: 2, acquisition time: 5 min 12 s), T1-weighted turbo spin echo in coronal plane (repetition time ms/echo time ms: 700/12; flip angle 150°, section thickness 2 mm; acquisition time: 1 min 47 s), proton density turbo spin echo with fat saturation in coronal plane (repetition time ms/echo time ms: 3000/38; flip angle 150°, section thickness 2 mm; acquisition time: 3 min 35 s), T1-weighted turbo spin echo with fat saturation in sagittal plane (repetition time ms/echo time ms: 682/15; flip angle 150°, section thickness 2 mm; acquisition time: 4 min 23 s), T2-trufi (TrueFISP) 3D in axial plane (repetition time ms/echo time ms: 11.69/4.89; flip angle 30°, section thickness 1 mm; acquisition time: 2 min 51 s), T2-trufi 3D in coronal plane (repetition time ms/echo time ms: 11.67/4.87; flip angle 30°, section thickness 1 mm; acquisition time: 1 min 48 s).

### Definition of ligaments

**Fig. 2:**
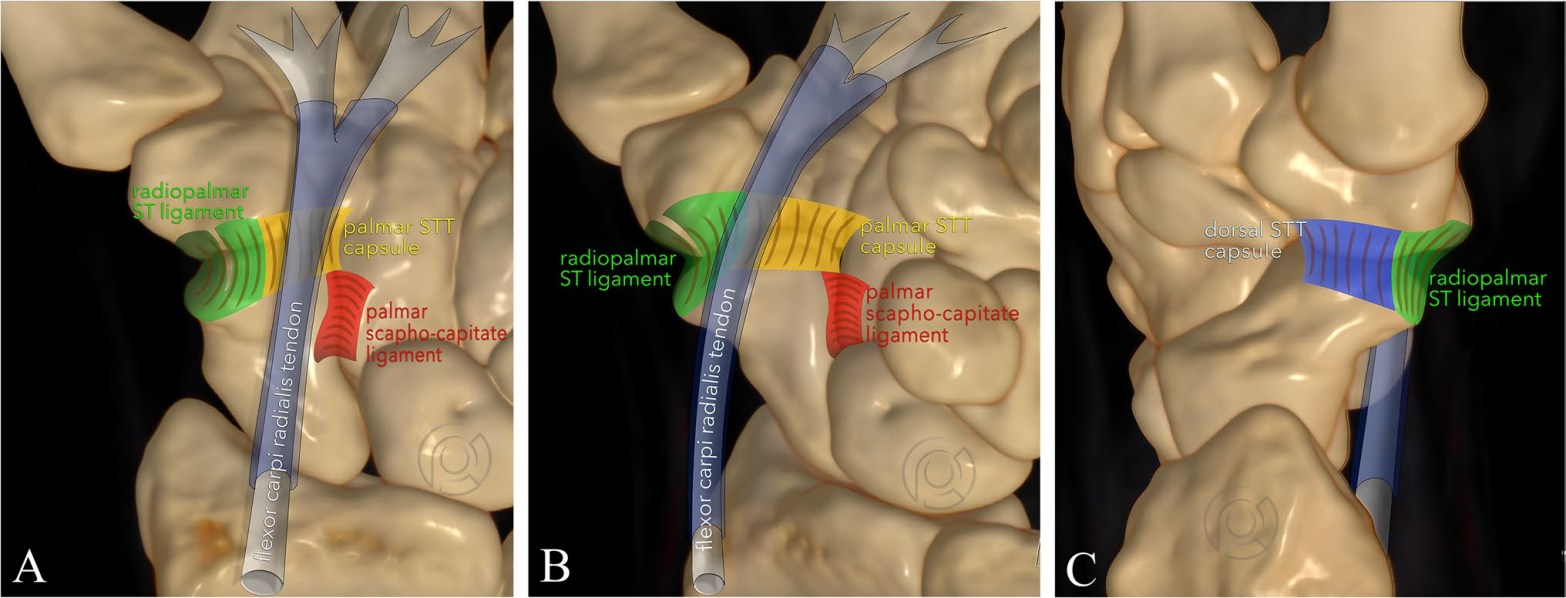
**a**–**c** Schematic illustration of the scapho-trapezial-trapezoidal ligament complex. Schematic illustration of the 4 components of the scapho-trapezial-trapezoidal ligament from different perspectives (**a**: from palmar, **b**: oblique view from ulnopalmar, **c**: lateral view from radial). Green: radiopalmar scapho-trapezial ligament (rpSTL), yellow: palmar scapho-trapezial-trapezoidal capsule (pSTTC), red: palmar scapho-capitate capsular ligament (pSCL), blue dorsal scapho-trapezial-trapezoidal capsule (dSTTC)

**Fig. 3:**
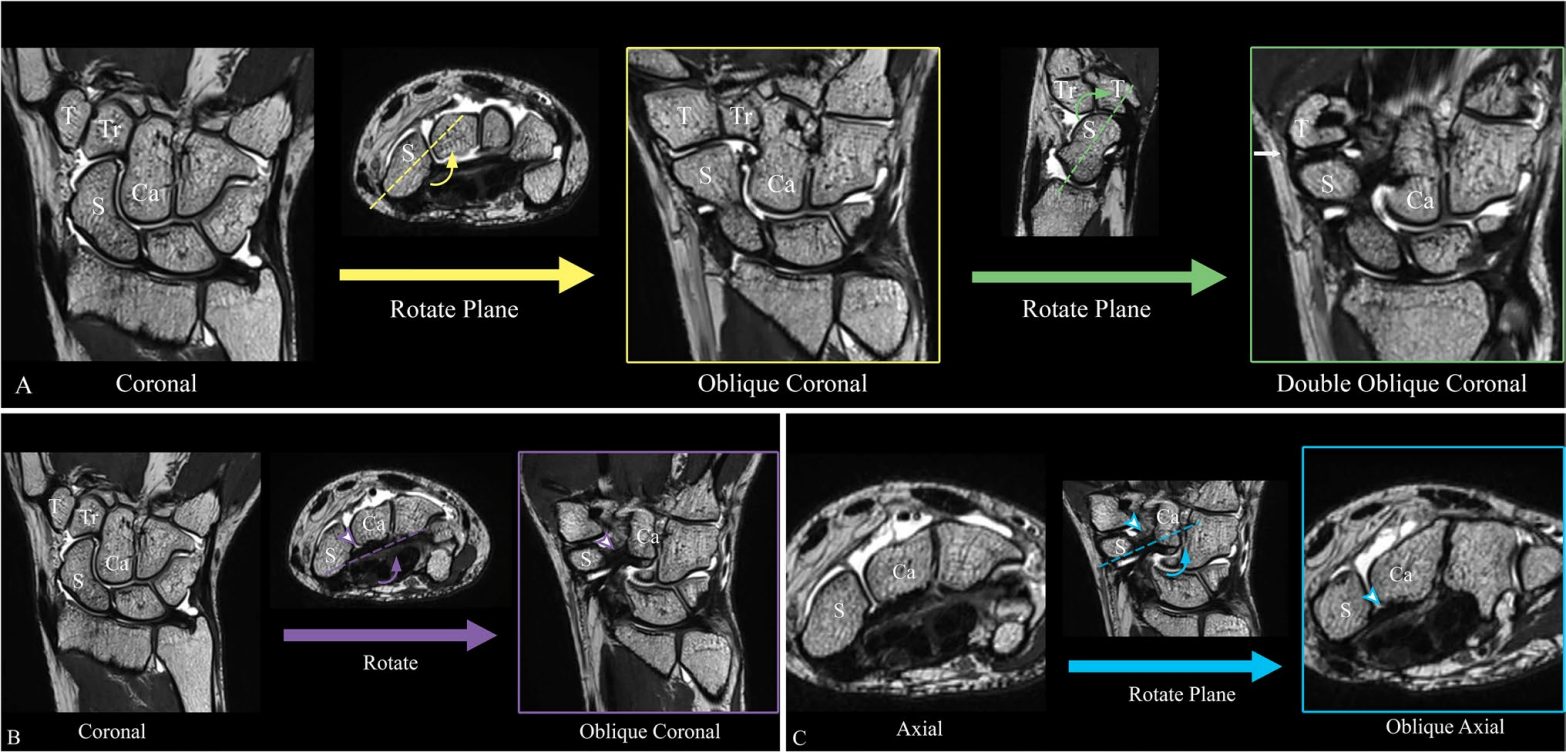
Illustrated image reconstruction manual for optimal depiction of the STT ligament complex. **a** Manual for the double oblique coronal view for the radiopalmar scapho-trapezial ligament (white arrow). Start off with a standard coronal view of the wrist, rotate (curved yellow arrow) the plane parallel to the long axis (in axial view) of the scaphoid (dashed yellow line), next tilt (green arrow) the plane parallel to the scaphoid (in sagittal view) (green dashed line). **b** Manual for the oblique coronal view of the scapho-capitate capsular ligament (white arrowhead). Start off with a standard coronal view of the wrist, rotate (curved purple arrow) the plane parallel to the scapho-capitate capsular ligament (dashed purple line) (in axial view). **c** Manual for the oblique axial view of the scapho-capitate capsular ligament (white arrowhead). Start off with a standard axial view of the wrist, rotate (curved blue arrow) the plane parallel to the scapho-capitate capsular ligament (dashed blue line) (in coronal view). Abbreviations: S, scaphoid; L, lunate; Tr, trapezoideum; Ca, capitate; T, trapezium

**Fig. 4:**
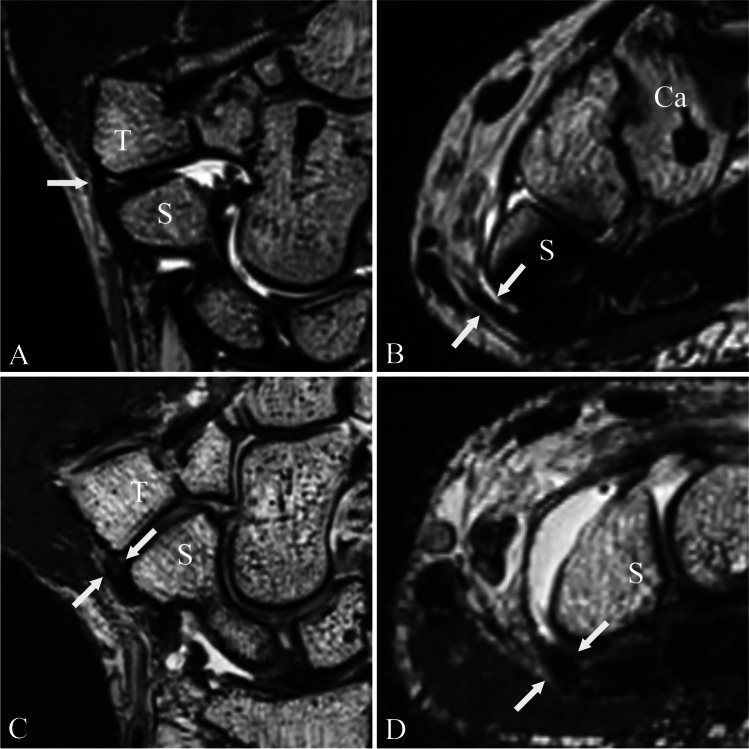
Radiopalmar scapho-trapezial ligament. Proton density-weighted (SPACE) images of a MR wrist arthrogram in coronal double oblique (**a** and **c**) (as shown in Fig. 3a) and axial plane (**b** and **d**). Images **a** and **b** show a normally appearing radiopalmar scapho-trapezial ligament (rpSTL) (white arrows) of a 31-year-old male asymptomatic patient. The rpSTL originates from the distal pol of the scaphoid and inserts into the ridge of the trapezoid. Note the normal low signal intensity of the ligament and the distinct morphology. Images **c** and **d** show the rpSTL (white arrows) of a 44-year-old male symptomatic patient with pain over the STT joint. Note the increased signal intensity and the indistinct ligament morphology of the rpSTL. Abbreviations: S, scaphoid; T, trapezium

**Fig. 5:**
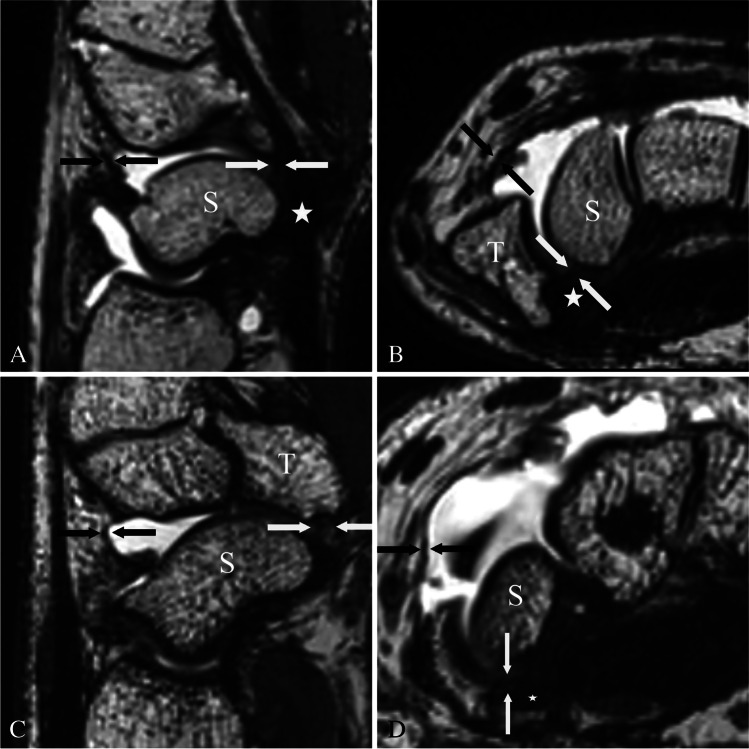
Palmar and dorsal scapho-trapezial-trapezoidal capsule. Proton density-weighted (SPACE) images of a MR wrist arthrogram in sagittal (**a** and **c**) and axial orientation (**b** and **d**). Images **a** and **b** show a normally appearing palmar (white arrows) and dorsal scapho-trapezial-trapezoidal capsule (black arrow) with normal signal intensity and morphology of a 30-year-old female asymptomatic patient. Images **c** and **d** show the palmar (white arrows) and dorsal scapho-trapezial-trapezoidal capsule (black arrow) of a symptomatic of a 36-year-old man with pain over the STT joint with the increased signal intensity and indistinct morphology. Note the proximity of the adjacent flexor carpi radialis tendon (white star). Abbreviations: S, scaphoid; T, trapezium

**Fig. 6:**
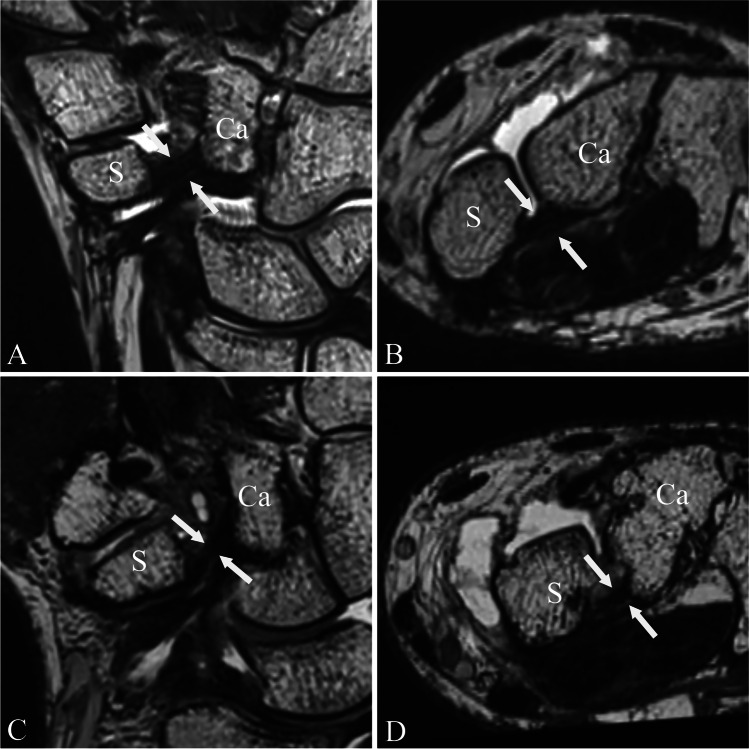
Palmar scapho-capitate capsular ligament. Proton density-weighted (SPACE) images of a MR wrist arthrogram in oblique coronal (**a** and **c**) and axial oblique orientation (**b** and **d**). Images **a** and **b** show the palmar scapho-capitate capsular ligament (pSCL; white arrows) of a 31-year-old male asymptomatic patient. The ligament shows normal low signal intensity and morphology. The pSCL connects the distal scaphoid pole with the capitate. Images **c** and **d** show the pSCL of a 42-year-old male symptomatic patient with pain over the STT joint. Note the increased signal intensity and indistinct ligament morphology of the pSCL (white arrows). Abbreviations: S, scaphoid; Ca, capitate

Four components of the STT ligament complex were identified. The rpSTL is located radial of the flexor carpi radialis (FCR) tendon and originates from the scaphoid tuberosity and inserts into the trapezial ridge (Fig. 2a–c). The ligament is best viewed and assessed in a coronal double oblique plane parallel to the long axis of the scaphoid (Figs. 3a,  4a–d). The pSTTC is located palmar to the rpSTL and forms the floor of the FCR tendon sheath. This structure is best viewed in sagittal orientation (Figs. 2a–c,  5a–d). The pSCL originates form the ulnar surface of the distal pole of the scaphoid and insert into the neck of the capitate. This ligament is best identified in coronal oblique plane parallel to the long axis of the scaphoid and in oblique axial plane (Figs. 2,  3b and c,  6a–d). The dSTTC forms a thin capsule and is best appreciated in axial orientation (Figs. 2,  5a–d).

### Image analysis

Image analysis of the scapho-trapezial-trapezoid ligament complex was independently performed by two fellowship-trained musculoskeletal radiologists with 6 (blinded) and 13 years (blinded) of experience in musculoskeletal radiology, respectively. All sequences were available for the analyses. Both readers were blinded to any clinical information and each other’s reading results.

### Qualitative analysis

For each component of the scapho-trapezial-trapezoid ligament complex following features were assessed: visibility (yes/no), signal intensity (low signal intensity, increased signal intensity, striated), morphology (distinct/indistinct). Low signal intensity and distinct morphology was interpreted as normal appearance. Additionally, the presence of contrast media in the tendon sheath of the flexor carpi radialis (yes/no) and distension of the STT joint with contrast media was assessed (no distension, moderate/suboptimal distension, good/optimal distension). Overall STT joint degeneration, bone marrow edema, osteophyte and chondral defects were assessed using a four-point scale (0 = none, 1 = mild, 2 = moderate, 3 = severe).

### Quantitative analysis

For each component of the scapho-trapezial-trapezoidal ligament complex, the thickness was measured at the midportion of each ligament. For the pSCL, additionally, the width of the ligament was measured in the coronal plane.

### Other causes for radial-sided wrist pain

Furthermore, a fellowship-trained musculoskeletal radiologist (blinded) who was not involved in the readout assessed other causes for radial-sided wrist pain based on the final MR report.

### Statistical analyses

Quantitative data are given as mean values and standard deviations, qualitative data as median and range. Cohen *κ* was calculated to assess the interreader agreement regarding the qualitative parameters. *κ* between 0.0 and 0.20 was defined as poor, between 0.21 and 0.4 as fair, between 0.41 and 0.60 as moderate, between 0.61 and 0.80 as good, and between 0.81 and 1.00 as excellent agreement. Intraclass correlation coefficient less than 0.69 was defined as poor, ICC between 0.70 and 0.79 as fair, ICC between 0.80 and 0.89 as good, and ICC greater than 0.9 as high. The Mann–Whitney *U* test was performed to compare demographics, qualitative and quantitative imaging features between asymptomatic and symptomatic group. A *p*-value of less than 0.05 was defined as statistically significant. For the statistical analyses, a commercially available software was used (SPSS, Version 26, Chicago/IL, USA).

## Results

During the inclusion period, a total of 60 MR arthrograms were performed. In 16 patients, no adequate pain assessment was reported in the medical history. Two patients were excluded due to bad image quality. In both cases, bad image quality was due to motion artifacts. Thus, a total of 42 consecutive patients, who underwent MR arthrogram of the wrist, were included in this study. Twenty-nine (69%) were male, 13 (31%) females. The median age was 37.5 years (range 18–70 years). Twenty-five of 42 (59.5%) study patients were asymptomatic: 17 (68.0%) men and 8 (32.0%) females with a median age of 34 years (range 19–61 years). Seventeen of 42 (40.5%) patients were classified as symptomatic: 12 (70.6%) men, 5 (29.4%) females with a median age of 44 years (range 18–70 years). Median time between clinical examination and MR arthrogram was 1 week. Between the asymptomatic and symptomatic group, no significant difference was observed in gender (*p* = 0.860) and age (*p* = 0.057).

### Qualitative analyses

A summary of the qualitative results by each reader is shown in Tables 1 and 2. The interreader agreement for the visibility of the rpSTL was good (*κ* = 0.659). The rpSTL was identified in 33 of 42 cases (78.6%) cases by both readers. In 3 cases, the rpSTL was only identified by reader 1 and in 1 case only by reader 2, respectively. In 5 of 42 cases (11.9%), no rpSTL was identified by both readers. Among those 33 cases identified by both readers, the interreader agreement for signal intensity and ligament morphology were excellent and good, respectively. Signal intensity was rated as normal in 22 of 33 (66.7%) and as increased in 10 of 33 (30.3%) cases by both readers and ligament morphology was rated distinct in 21 of 33 cases (63.3%) and indistinct in 9 of 33 cases (27.3%) by both readers. Disagreement was observed in 3 of 33 cases (3%) for signal intensity and ligament morphology.

**Table 1:** Qualitative characteristics of the ligaments of the STT joint asymptomatic (*n* = 25) and symptomatic (*n* = 17) patients

		Ligament	Visibility	Ligament signal intensity			Ligament morphology	
				Low	Increased	Striated	Distinct	Indistinct
Asymptomatic	Reader 1	rpSTL	20/25 (80.0%)	16/20 (80.0%)	4/20 (20%)	0/20(0%)	14/20 (70.0%)	6/20 (30.0%)
		pSTTC	14/25 (56.0%)	12/14 (85.7%)	2/14 (14.3%)	0/14 (0%)	12/14 (85.7%)	2/14 (14.3%)
		pSCL	25/25 (100%)	12/25 (48.0%)	6/25 (24.0%)	7/25 (28.0%)	23/25 (92.0%)	2/25 (8.0%)
		dSTTC	25/25 (100%)	24/25 (96.0%)	1/25 (4.0%)	0/25 (0%)	23/25 (92.0%)	2/25 (8.0%)
	Reader 2	rpSTL	20/25 (80.0%)	17/20 (85.0%)	3/20 (15.0%)	0/20 (0%)	16/20 (80.0%)	4/20 (20%)
		pSTTC	12/25 (48.0%)	12/12 (100%)	0/12 (0%)	0/12 (0%)	12/12 (100%)	0/12 (0%)
		pSCL	25/25 (100%)	11/25 (44.0%)	8/25 (32.0%)	6/25 (24.0%)	20/25 (80.0%)	5/25 (20.0%)
		dSTTC	25/25 (100%)	23/25 (92.0%)	2/25 (8.0%)	0/25 (0%)	21/25 (84.0%)	4/25 (16.0%)
Symptomatic	Reader 1	rpSTL	16/17 (94.2%)	7/16 (43.8%)	9/16 (56.3%)	0/16 (0%)	8/16 (50.0%)	8/16 (50.0%)
		pSTTC	8/17 (47.1%)	4/8 (50.0%)	4/8 (50.0%)	0/8 (0%)	5/8 (62.5%)	3/8 (37.5%)
		pSCL	17/17 (100%)	6/17 (35.3%)	10/17 (58.8%)	1/17 (5.9%)	11/17 (64.7%)	6/17 (35.3%)
		dSTTC	17/17 (100%)	15/17 (88.2%)	2/17 (11.8%)	0/17 (0%)	15/17 (88.2%)	2/17 (11.8%)
	Reader 2	rpSTL	14/17 (82.4%)	7/14 (50.0%)	7/14 (50.0%)	0/14 (0%)	9/14 (64.3%)	5/14 (35.7%)
		pSTTC	6/17 (35.3%)	4/6 (66.7%)	2/6 (33.3%)	0/6 (0%)	5/6 (83.3%)	1/6 (16.7%)
		pSCL	17/17 (100%)	7/17 (41.2%)	9/17 (52.9%)	1/17 (5.9%)	11/17 (64.7%)	6/17 (35.3%)
		dSTTC	17/17 (100%)	15/17 (88.2%)	2/17 (11.8%)	0/17 (0%)	13/17 (76.5%)	4/17 (23.5%)

*Abbreviations*: *rpSTL*, radiopalmar scapho-trapezial ligament; *pSTTC*, palmar scapho-trapezial-trapezoidal capsule; *pSCL*, palmar scapho-capitate-capsular ligament; *dSTTC*, dorsal scapho-trapezial-trapezoidal capsule

**Table 2:** Interreader agreement for the qualitative image criteria

Ligament	Visibility	Signal intensity	Morphology
rpSTL	0.659	0.930	0.792
pSTTC	0.716	0.491	0.341
pSCL	1.000	0.887	0.797
dSTTC	1.000	0.844	0.618

*Abbreviations*: *rpSTL*, radiopalmar scapho-trapezial ligament; *pSTTC*, palmar scapho-trapezial-trapezoidal capsule; *pSCL*, palmar scapho-capitate-capsular ligament; *dSTTC*, dorsal scapho-trapezial-trapezoidal capsule

The interreader agreement for the identification of the pSTTC was good (*κ* = 0.716). The pSTTC was only identified in 17 of 42 (40.5%) cases by both readers. In 19 of 42 cases (45.2%), no pSTTC could be identified by either of the readers. In 5 cases, the pSTTC was only identified by reader 1 and in 1 case only by reader 2 respectively. Among those cases identified by both readers, signal intensity was rated normal in 12 of 17 (70.6%) and increased in 2 of 17 (11.8%) cases and ligament morphology was rated as distinct in 13 of 17 (76.5%) and as indistinct in 1 of 17 (5.9%) cases by both readers. Disagreement was observed in 3 of 17 cases (17.6%) for signal intensity and 3 of 17 cases (17.6%) ligament morphology.

The pSCL was identified in all cases by both readers. The interreader agreement for signal intensity and ligament morphology were excellent and good respectively. In 17 of 42 cases (40.5%), the signal intensity was rated as normal, in 15 of 42 cases (35.7%) as increased and in 7 of 42 cases (16.7%) as striated by both readers. The morphology of the ligament was rated as distinct in 31 of 42 cases (73.9%) and in 8 of 42 cases (19%) by both readers. Disagreement was observed in 3 of 42 cases (7.1%) for signal intensity and 3 of 42 cases (7.1%) ligament morphology.

The dSTTC was also identified in all cases by both readers. The interreader agreement was good for signal intensity and fair for ligament morphology. In 38 of 42 cases (90.5%), the signal intensity was rated as normal, in 3 of 42 cases (7.1%) as increased. In 34 of 42 (81.0%), cases the morphology was rated as distinct and in 4 cases (9.5%) as indistinct. Disagreement was observed in 1 of 42 cases (2.4%) for signal intensity and 4 of 42 cases (9.5%) ligament morphology.

Contrast media in the tendon sheath of the flexor carpi radialis tendon was observed in 3 cases by both readers. STT joint distention with contrast media was rated as not distended in 2 of 42 cases (4.8%), moderately distended in 10 of 42 cases (23.8%) and well distended in 28 of 42 cases (66.7%). Overall joint degeneration was rated as absent in 32 and 33 cases, little in 7 and 5 cases and moderate in 3 and 4 cases by readers 1 and 2, respectively. No extensive joint degeneration was observed. Bone marrow edema was rated as absent in 34 and 34, little in 7 and 6, moderate in 1 and 2 and extensive in 0 and 0. Osteophyte was rated as absent in 37 and 36, little in 4 and 5, moderate in 1 and 1 and extensive in 0 and 0. Chondral defects were rated as absent in 32 and 33, little in 6 and 5, moderate in 2 and 1, and extensive in 3 and 3. Image quality rated as good in 35 and 36 and as impaired in 7 and 6 cases by readers 1 and 2.

### Quantitative analyses

A summary of the quantitative analyses is given in Table 3. Intraclass correlation coefficients were good for all measurements. Mean thickness of the rpSTL was 1.4 ± 0.5 mm and 1.3 ± 0.5 mm for readers 1 and 2 with an ICC value of 0.878. Mean thickness of the pSTTC was 0.5 ± 0.5 mm and 0.4 ± 0.4 mm for readers 1 and 2 with an ICC value of 0.865. Mean thickness of the pSCL was 2.8 ± 0.7 mm and 2.7 ± 0.6 mm for readers 1 and 2 with an ICC value of 0.809 and the mean width of the ligament was 3.5 ± 0.7 mm 3.3 ± 0.6 mm for readers 1 and 2 with an ICC value of 0.838. Mean thickness of the dSTTC was 0.5 ± 0.3 mm and 0.3 ± 0.3 mm for readers 1 and 2 with an ICC value of 0.810.

**Table 3:** Thickness of the ligaments of the STT joint in asymptomatic (*n* = 25) and symptomatic (*n* = 17) patients

	Ligament	Reader 1	Reader 2
Asymptomatic	rpSTL	1.3 ± 0.5 mm	1.2 ± 0.5 mm
	pSTTC	0.5 ± 0.5 mm	0.4 ± 0.4 mm
	pSCL axial	2.8 ± 0.7 mm	2.8 ± 0.6 mm
	pSCL coronal	3.5 ± 0.5 mm	3.4 ± 0.5 mm
	dSTTC	0.5 ± 0.4 mm	0.3 ± 0.3 mm
Symptomatic	rpSTL	1.5 ± 0.5 mm	1.4 ± 0.4 mm
	pSTTC	0.5 ± 0.4 mm	0.3 ± 0.3 mm
	pSCL axial	2.9 ± 0.7 mm	2.7 ± 0.6 mm
	pSCL coronal	3.5 ± 0.9 mm	3.2 ± 0.8 mm
	dSTTC	0.5 ± 0.3 mm	0.4 ± 0.2 mm

*Abbreviations*: *rpSTL*, radiopalmar scapho-trapezial ligament; *pSTTC*, palmar scapho-trapezial-trapezoidal capsule; *pSCL*, palmar scapho-capitate-capsular ligament; *dSTTC*, dorsal scapho-trapezial-trapezoidal capsule

### Comparison between asymptomatic and symptomatic group

A summary of the results is given in Table 4. Increased signal of the rpSTL was more commonly observed in the symptomatic group (*p* = 0.027 for reader 1 and *p* = 0.030 for reader 2). No significant difference was observed in ligament morphology (*p* = 0.228 for reader 1 and *p* = 0.314 for reader 2). Reader 1 observed a significant difference in ligament morphology in the pSCL (*p* = 0.029), whereas reader 2 observed no significant difference (*p* = 0.274). For all other ligaments, no significant difference was observed in signal intensity and morphology. For the quantitative analyses, no significant difference was observed in the ligament thickness between the asymptomatic and symptomatic groups (*p*-values ranging between 0.095 and 0.990) (Table 4). Contrast media in the tendon sheath of the flexor carpi radialis tendon was observed in two cases in the symptomatic group and one case in the asymptomatic group. Joint degeneration was significantly more often observed in the symptomatic group by both readers (*p* = 0.003 for reader 1 and *p* = 0.012 for reader 2). Joint degeneration was observed in only two cases by both readers in the asymptomatic group and 8 and 7 cases in the symptomatic group by readers 1 and 2 respectively.

**Table 4:** Comparison of quantitative image characteristics between asymptomatic (*n* = 25) and symptomatic (*n* = 17) patients based on Table 1

	Ligament	Reader 1 *p*-value	Reader 2 *p*-value
Qualitative analyses	rpSTL visibility	0.205	0.851
	rpSTL intensity	0.027	0.030
	rpSTL morphology	0.228	0.314
	pSTTC visibility	0.574	0.420
	pSTTC signal intensity	0.188	0.291
	pSTTC morphology	0.402	0.616
	pSCL visibility	1.000	1.000
	pSCL signal intensity	0.890	0.637
	pSCL morphology	0.029	0.274
	dSTTC visibility	1.000	1.000
	dSTTC signal intensity	0.343	0.687
	dSTTC morphology	0.687	0.547
	Degeneration of the STT joint	0.003	0.012
Quantitative analyses	rpSTL thickness	0.337	0.300
	pSTTC thickness	0.664	0.682
	pSCL thickness axial	0.768	0.990
	pSCL thickness coronal	0.672	0.223
	dSTTC thickness	0.907	0.095

*Abbreviations*: *rpSTL*, radiopalmar scapho-trapezial ligament; *pSTTC*, palmar scapho-trapezial-trapezoidal capsule; *pSCL*, palmar scapho-capitate capsular ligament; *dSTTC*, dorsal scapho-trapezial-trapezoidal capsule; *STT*, scapho-trapezio-trapezoidal

### Other causes for radial-sided wrist pain

Alternative causes for radial-sided wrist pain (apart from the STT joint and its ligaments) were found as follows: Abnormality of the scapho-lunate ligament was reported in 12 of 25 (48%) patients in the asymptomatic group and in 10 of 17 (58.8%) patients in the symptomatic group. Radial-sided ganglion was detected in 5 of 25 (20%) patients in the asymptomatic group and in 2 of 17 (11.8%) patients in the symptomatic group. No bone contusions were reported in any groups. Between the asymptomatic and symptomatic group, no significant difference was observed in frequency of scapho-lunate ligament abnormality (*p* = 0.496) or ganglion (*p* = 0.487).

## Discussion

The STT ligament complex acts as a stabilizer of the STT joint [8]. Injuries of the STT ligament complex are rare but might lead to pain over the STT joint, chronic instability, and decrease of thumb-index-middle finger pinch strength [1]. Isolated injuries of the STT ligament complex are rare but can be treated surgically by reconstruction or suture of the torn ligament components [1]. If an adequate ligament remnant can be found, typical lesion at the most palmar side of the STT joint (up to 1 cm length), involving the rpSTL and the palmar joint capsule, can be closed with several sutures. Furthermore, the flexor carpi radialis tendon can be sutured to the tubercle of scaphoid to protect and to reinforce the ligament repair[10]. If the remnant of the ligament is not of an adequate quality, a reconstruction with a tendon transplant (e.g. palmaris longus) can be performed. The tendon is pulled through a transverse V-shaped drill hole at the trapezium and tubercle of the scaphoid and typically fixed in a O-configuration.

In our study, we were able to outline all four components of the STT ligament complex using a 3D high-resolution 3 T MR sequence. The rpSTL was visible in almost all cases with a good interreader agreement. The ligament was best visualized using a double oblique coronal plane nearly parallel to the long axis of the scaphoid (Fig. 3a). Using this double oblique coronal orientation, we were able to visualize the entire diagonal course of the ligament from its origin at the distal pole of the scaphoid to the trapezial ridge. The ligament showed an average thickness of 1.3 ± 0.5 mm. The biomechanical function of the STT ligament complex is not well understood yet, however, the rpSTL ligament has been described as the main stabilizer [8]. Being the second thickest ligament in the STT ligament complex underlines its role as an important stabilizer of the STT joint. The ligament showed variable signal intensity, with the majority of cases showing a normal low signal intensity. Interestingly, in the symptomatic group, the signal intensity of the ligament was rated as increased significantly more frequently by both readers as compared to the asymptomatic group, indicating that increased signal intensity might be a sign of ligament degeneration or posttraumatic changes. It remains unclear, why the rpSTL could not have been visualized in all cases. Holveck et al. described the ST ligament as a V-shaped ligament consisting of a thicker ulnar and a thinner radial slip [3]. In our study, we were only able to reliably visualize the ulnar slip as a distinct structure but not the radial slip. It might be explained by the close proximity to the adjacent dorsal scapho-trapezio-trapezial capsule and the smaller thickness of the radial slip. Also, the significance of non-visualization of the rpSTL remains unclear, especially as interreader agreement of ligament visibility was only rated as good (*κ* = 0.659). However, the frequency of ligament visibility was similar in the asymptomatic and symptomatic group. Moreover, no significant difference in terms of visibility of the rpSTL between the asymptomatic and symptomatic groups was observed.

The pSTTC was only visible in approximately 50% of cases. It was best visible in the sagittal orientation as a thin structure connecting the scaphoid with the trapezium radial of the FCR tendon sheath with an average thickness of 0.4 ± 0.4 mm. As described before, part of the palmar scapho-trapezial-trapezoidal capsule contributes to the floor of the tendon sheath of the flexor carpi radialis tendon and was therefore not always clearly distinguishable from the FCR tendon sheath and visible as a distinct ligamentous structure [8]. Being a thin structure and hard to distinguish from the adjacent FCR tendon might explain the moderate and fair interreader agreement for signal intensity and morphology. In three cases, contrast media in the tendon sheath of the FCR was observed which has been described in the literature as an indirect sign for an injury of the palmar scapho-trapezial-trapezoidal capsule [1]. Two of these cases were observed in the symptomatic group, where one patient had a recent wrist trauma and the other patient showed moderate STT joint degeneration. Despite being a thin structure with a thickness of only 0.5 mm of thickness, the dorsal capsule was identified in all study patients. This might be explained by the well-distended joint with contrast media and the adjacent subcutaneous fat tissue.

The pSCL was visible in all study subjects. Similarly, to the rpSTL ligament it was best visualized in an oblique coronal orientation nearly parallel to the long axis of the scaphoid and axial orientation. The pSCL originated form the ulnar surface of the distal pole of the scaphoid and insert into the neck of the capitate and ran parallel and in close proximity to the adjacent radio-scapho-capitate ligament which originated from the styloid process of the radius [6]. The ligament was the thickest of the STT ligament complex with a mean thickness of 2.8 ± 0.8 mm. This ligament showed a high variance in terms of SI with the majority of the ligament showing a low signal intensity. However, in approximately 20% of cases, the ligament showed a striated appearance.

In our institution we implemented a proton density-weighted 3D sequence with a voxel size of 0.2 × 0.2 × 0.5 mm into our MRI standard protocol of the wrist on our 3 T device. The acquisition time of this sequence is 5 min and 12 s and the whole protocol 19 min and 36 s. Using this sequence, we were able to identify all four components of the STT ligament complex, most importantly the rpSTL, which would have been difficult to visualize in conventional sequences due to its diagonal course and decreased thickness (2–3 mm).

The following limitations must be acknowledged. First, the retrospective nature of this study with its inherent shortcoming. Second, the small number of only 42 study patients. Third, the distinction into asymptomatic and symptomatic group was solely based on the symptoms reported by the patients in the medical history. No distinctions were made in terms of possible trauma or trauma mechanism. Fourth, the influence of joint distention for the delineation of the STT ligament complex could not be assessed due to the small sample size. Moreover, 3D SPACE was only performed after contrast media injection. Comparison of ligament delineation of 3D SPACE between non-arthrogram MRI and MR arthrogram or the degree of joint distension would have been interesting.

The anatomy of the STT ligament complex can consistently be visualized on high-resolution 3D MRI. Morphologic changes of radiopalmar scapho-trapezial ligament are significantly more common in patients with radial-sided wrist pain.
